# Pharmacotherapy and cardiovascular challenges: a case report of olverembatinib-induced myocardial infarction with non-obstructive coronary arteries

**DOI:** 10.1186/s12872-024-04011-w

**Published:** 2024-07-02

**Authors:** Haiyan Xue, Lan Wang, Yuliang Ma, Chang Hou

**Affiliations:** 1 Department of Critical Care Medicine, Peking University People’s Hospital, Beijing, China; 2 Department of Cardiology, Peking University People’s Hospital, Beijing, China; 3 Beijing Key Laboratory of Early Prediction and Intervention of Acute Myocardial Infarction, Peking University People’s Hospital, Beijing, China

**Keywords:** MINOCA, Olverembatinib, TKI

## Abstract

The anticancer drug of tyrosine kinase-inhibitors (TKIs) has significantly improved the prognosis of patients with specific leukemia but has also increased the risk of organ adverse reactions. Herein, we present a case of a patient diagnosed with myeloproliferative neoplasms who experienced recurrent chest pain after receiving treatment with Olverembatinib. Electrocardiography and coronary angiography confirmed the diagnosis of myocardial infarction with non-obstructive coronary arteries. This case serves as a reminder for clinicians to pay more attention and actively prevent the cardiac adverse reactions of TKIs when using such medications.

## Introduction

The introduction of tyrosine kinase inhibitors (TKIs) has revolutionized the treatment of various hematologic malignancies, notably improving the prognosis and overall survival rates for patients with specific types of leukemia, including myeloproliferative neoplasms (MPNs) [1, 2]. TKIs, such as Olverembatinib, target aberrant signaling pathways critical for the proliferation and survival of malignant cells, providing a targeted therapeutic approach that has significantly improved clinical outcomes [3]. However, the therapeutic success of TKIs is often accompanied by a spectrum of adverse effects, particularly concerning their impact on cardiovascular health [4]. Emerging evidence has indicated that TKIs can precipitate serious cardiovascular events, including myocardial infarction (MI), even in the absence of significant coronary artery obstruction [5–8]. This phenomenon, known as myocardial infarction with non-obstructive coronary arteries (MINOCA), represents a diagnostic and therapeutic challenge, underscoring the need for comprehensive cardiac monitoring and preventive strategies in patients undergoing TKIs therapy [9]. In this report, we present a case of a patient with MPN who experienced recurrent chest pain and was subsequently diagnosed with MINOCA after receiving Olverembatinib. This case highlights the importance of vigilance and proactive management of potential cardiac adverse effects in patients treated with TKIs. By detailing this case, we aim to raise awareness among clinicians regarding the cardiovascular risks associated with TKI therapy and emphasize the necessity of integrating cardiac safety concerns in the management of patients receiving these potent anticancer agents.

## Case presentation

A 61-year-old male with a one-year history of well-controlled hypertension managed with felodipine, a one-pack-year smoking history (had been successfully quit for the past six months), and a body mass index of 22.49 kg/m^2^, without any other risk factors for atherosclerosis, was admitted to our cardiac care unit due to acute MI. Four months prior to admission, the patient had been diagnosed with myeloid neoplasm with FGFR1 gene rearrangement and polycythemia vera, for which he had been undergoing treatment with Olverembatinib at a dose of 40 mg every other day for four months.

**Fig. 1:**
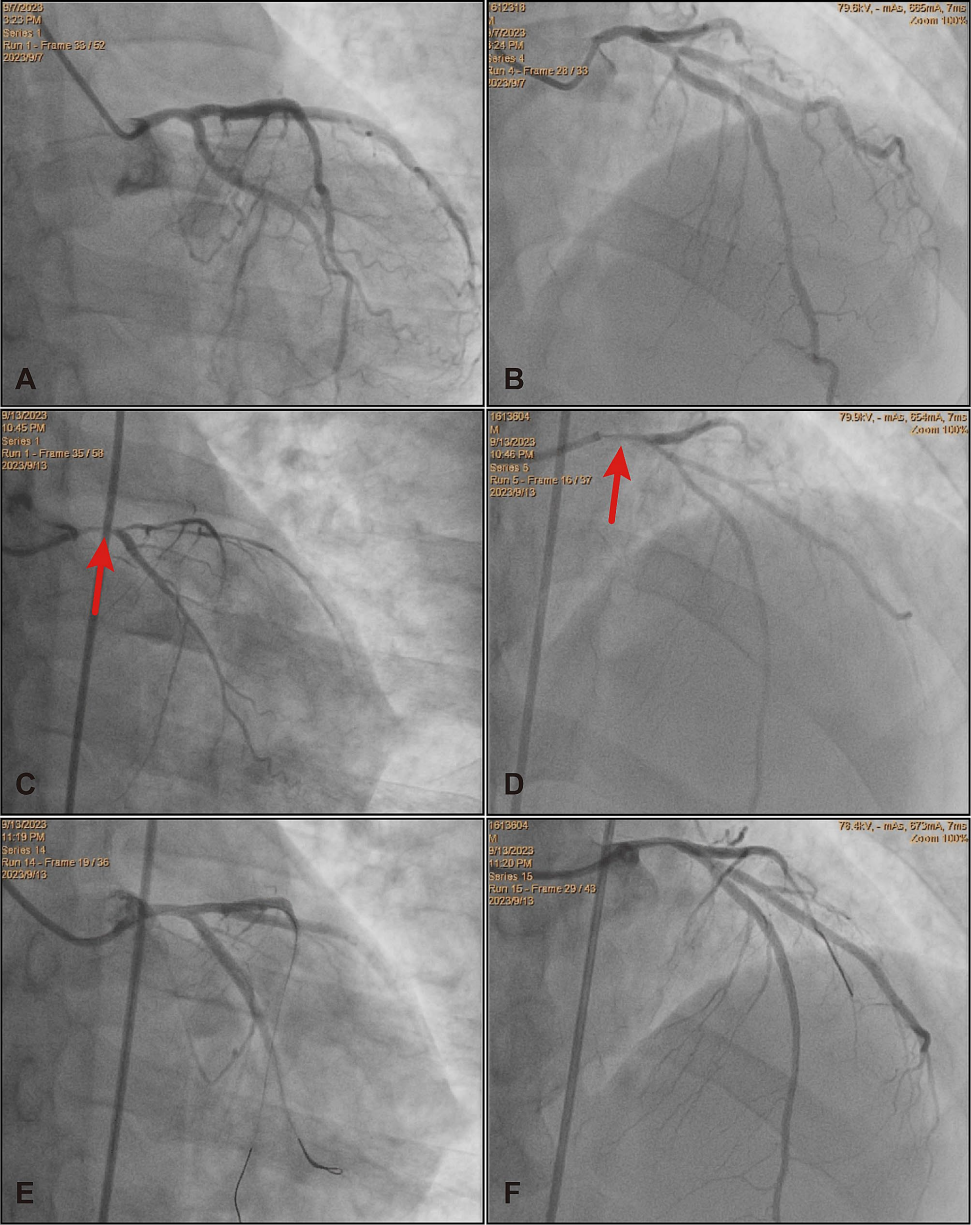
Coronary angiography images performed at the baseline and during coronary artery spasm. **A-B**. Mild stenosis in left main and proximal-left anterior descending artery as well as myocardial bridge in mid-left anterior descending artery were found on the baseline coronary angiography images. **C-D**. Diffuse spasm was observed especially at the left main and proximal-left anterior descending artery (labelled by the arrow) on the urgent coronary angiography. **E-F**. After the emergent balloon angioplasty, there was no significant stenosis and the coronary blood flow became normal

**Fig. 2:**
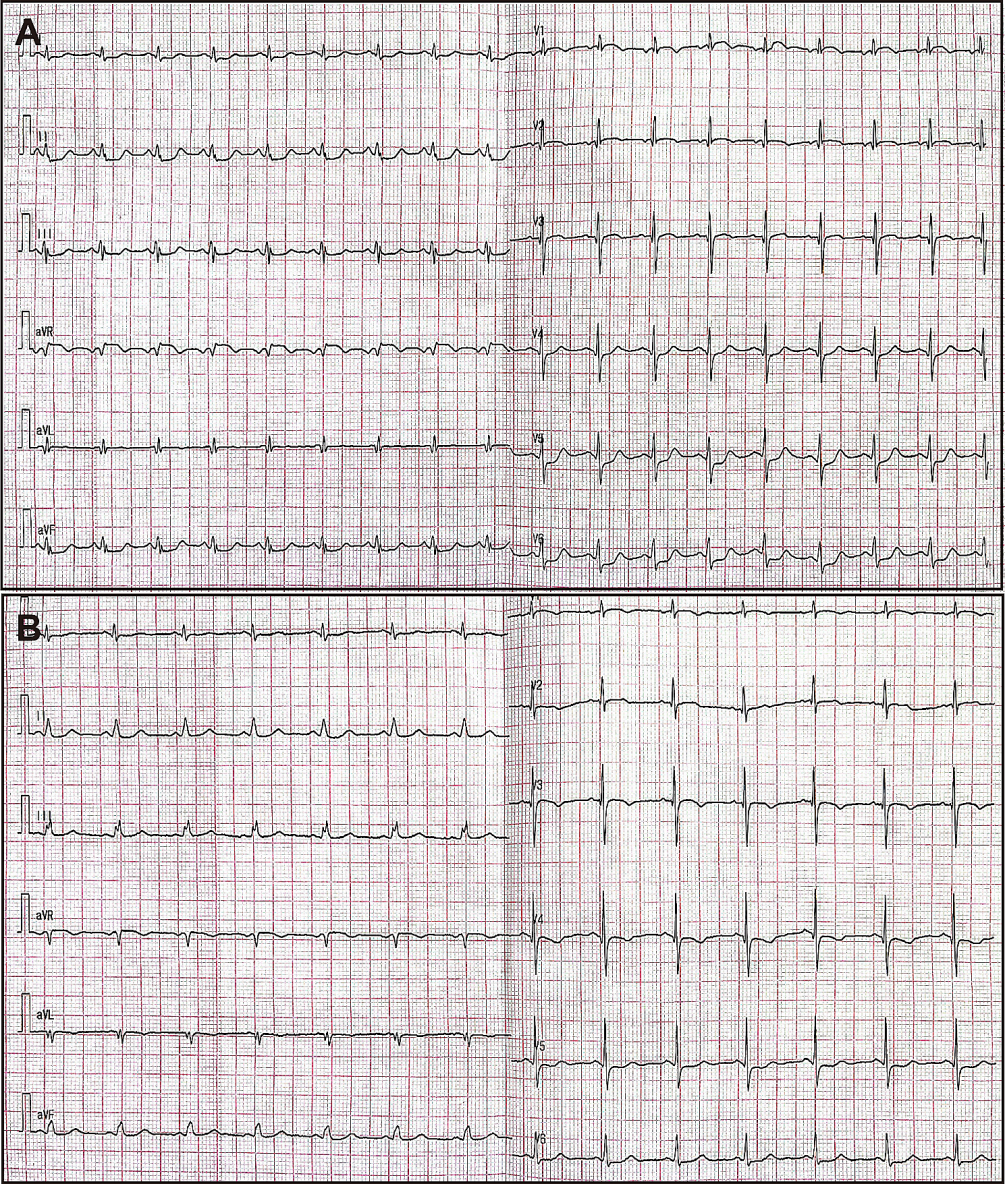
Electrocardiogram during the episodes and resolution of coronary artery spasm. **A**. Electrocardiogram showed ST-segment depression in multiple leads, along with ST-segment elevation in lead aVR during the attack of coronary artery spasm. **B**. The ST-segment deviation on electrocardiogram returned to normal during the resolution of coronary artery spasm

The patient began experiencing exertional chest discomfort accompanied by decreased exercise capacity in August 2023, without receiving any specific treatment. The values of the white blood cell and platelet count were initially within the normal range. Coronary angiography which was performed on September 7, 2023 revealed mild stenotic lesions (approximately 30–40% of diameter stenosis) in left main and proximal segment of left anterior descending artery (Fig. 1A and B). The levels of cardiac biomarkers, including creatine kinase MB isoform and cardiac troponin I, were within the normal range. However, six days later, the patient’s symptoms worsened. He developed persistent and refractory chest pain, and during the episodes, the 12-lead electrocardiography displayed ST-segment depression in leads II, III, aVF, and V4-V6, along with ST-segment elevation in lead aVR, which returned to normal when the symptom relieved (Fig. 2A and B). Additionally, the levels of cardiac biomarkers increased (creatine kinase MB isoform 10ng/ml (reference range: 0.5-5.0ng/ml) and cardiac troponin I 5.5ng/ml (reference range: 0.00-0.40ng/ml)) dramatically. Therefore, an urgent coronary angiography was repeated using the 6 F JL 4.0 diagnostic catheter, which showed severe stenosis in left main (Fig. 1C and D) and completely normal right coronary artery. After the intracoronary bolus of nitroglycerin, the stenosis attenuated greatly indicating the occurrence of coronary artery spasm. Subsequently, the patient underwent emergent balloon angioplasty of left main and proximal segment of left anterior descending artery, to treat the residual stenosis and further improve the coronary blood flow (balloon size: 2.00 mm in diameter, inflation pressure: 8 atm) (Fig. 1E and F). Following the procedure, the patient received intravenous administration of nitroglycerin, diltiazem, and oral aspirin. Consequently, the patient demonstrated symptom improvement without any recurrence of chest pain. Meanwhile, the levels of cardiac troponin I gradually declined to within the normal range. Concomitant with these events, the patient demonstrated a progressive elevation in the white blood cell count, reaching 27,310/µl (reference range: 3500–9500/µl). Thus, the administration of Olverembatinib was ceased, and the patient was finally transferred to the hematology department for additional therapeutic interventions, with the consideration of probable leukemic transformation.

## Discussion

This report presents the first documented case of MINOCA resulting from coronary artery spasm following treatment with Olverembatinib, which is one kind of third-generation TKI. Several cases of MINOCA during treatment with other second-generation TKIs have been previously reported, raising concerns about vascular toxicity and adverse vascular events in patients receiving such therapies [6–8]. Olverembatinib has largely addressed the issue of chronic myeloid leukemia (CML) resistance to second-generation TKIs, but adverse events may still occur. The potential mechanisms underlying Olverembatinib-induced coronary artery spasm remain unclear. It is hypothesized that various factors may contribute to this process, including autonomic nervous system alterations and drug-induced vascular toxicity. Additionally, the patient experienced probable leukemic transformation concomitant with the MI event, suggesting that leukocytosis and inflammation-induced vascular spasm should also be considered.

Although coronary artery spasm is a rare adverse event, its consequences can be severe, including myocardial ischemia, MI, cardiac arrest, and even sudden death [10]. Diagnostic challenges arise due to the possibility of coronary artery spasm occurring without evident stenosis. Therefore, timely recognition and effective treatment of coronary artery spasm are crucial to prevent disease progression. In the present case, the electrocardiography showes diffuse depression with ST-segment elevation in lead aVR during the episode of chest pain, which is consistent with the left main lesion. Therefore, despite considering the stenosis is mainly due to spasm, we still perform the angioplasty for this patient.

Potential drug-drug interactions involving Olverembatinib must also be considered. In this case, post-angioplasty medical treatment included the use of nitroglycerin, diltiazem, and aspirin. The combined use of these medications requires careful consideration of potential interactions. For instance, diltiazem is a CYP3A4 inhibitor and could increase the levels of Olverembatinib, thereby exacerbating its cardiovascular effects [11]. Utilizing physiologically based pharmacokinetic models could provide insights into these interactions by simulating and predicting the drug’s absorption, distribution, metabolism, and excretion within the body [11]. It’s also helpful to understand how Olverembatinib interacts with other medications or substances within the body, potentially influencing its safety and efficacy profile.

Given the patient’s hemoglobin level, the potential role of polycythemia and hypertension in the symptom of exertional angina should also be considered [12]. Furthermore, stress hyperglycemia has been known to drive the risk of hospitalization for chest pain in patients with ischemia with non-obstructive coronary arteries, adding another layer of complexity in managing these patients [13]. Other TKIs, such as Nilotinib, have been found to associate with accelerated atherosclerosis. This is relevant to the discussion because exertional chest discomfort is not typically indicative of coronary artery spasm. Studies have shown Nilotinib-associated atherosclerosis presenting as multifocal intracranial stenosis and acute stroke, as well as progressive peripheral arterial occlusive disease and other vascular events during Nilotinib therapy in CML patients [14, 15]. These findings underscore the importance of considering coronary artery atherosclerosis in the differential diagnosis. Advanced imaging modalities, such as intravascular ultrasound (IVUS), may provide more detailed insights into the underlying vascular pathology.

This study has several strengths, including detailed clinical observation and documentation of a rare adverse event associated with Olverembatinib. The report contributes to the limited body of literature on cardiovascular risks associated with TKI therapy, emphasizing the need for close cardiac monitoring. However, the study also has several limitations. The primary limitation is the inability to definitively establish causality between Olverembatinib treatment and the coronary artery spasm. The observational nature of the report inherently makes it difficult to control for potential confounders. Potential biases include patient selection bias, as this is a single-case report, and the possibility of other unadjusted factors contributing to the cardiovascular event. It is important to note that other causes, such as smoking, genetic predisposition or catheter irritation, might have been responsible for the vasospasm, although cocaine abuse can be ruled out in this patient. Secondly, the IVUS examination is not performed during the procedure. Therefore the formation of thrombus in the left main as the possible etiology of MI can not be completely excluded, even if there is no evidence of thrombi on the coronary angiography.

To mitigate the risks of cardiovascular events in patients receiving TKI therapy, physicians should take several proactive measures. Close monitoring of cardiac function, regular cardiovascular assessments, and the use of preventive medications such as antispasmodics or long-acting oral nitrates are essential. Identifying and managing other risk factors, such as hypertension and hyperglycemia, are also critical in the prevention of adverse outcomes.

In conclusion, while this case highlights an important potential adverse effect of Olverembatinib, further research is necessary to fully understand the mechanisms and risk factors involved in this process. Close monitoring and a multidisciplinary team approach are essential for managing the patients on TKI therapy to reduce potential cardiovascular risks.

## Data Availability

No datasets were generated or analysed during the current study.
